# Probiotics and prebiotics in clinical tests: an update

**DOI:** 10.12688/f1000research.19043.1

**Published:** 2019-07-22

**Authors:** Harald Brüssow

**Affiliations:** 1 Laboratory of Gene Technology, Department of Biosystems, KU Leuven University, Kasteelpark Arenberg 21, Leuven, 3001, Belgium

**Keywords:** probiotic, prebiotic, synbiotic, Lactobacillus, Bifidobacterium, diarrhea, sepsis

## Abstract

Probiotics have been explored in an exponentially increasing number of clinical trials for their health effects. Drawing conclusions from the published literature for the medical practitioner is difficult since rarely more than two clinical trials were conducted with the same probiotic strain against the same medical condition. Consequently, the European Society for Paediatric Gastroenterology, Hepatology and Nutrition (ESPGHAN) made a few recommendations restricting it to probiotic use against acute gastroenteritis and antibiotic-associated diarrhea. Recent studies also made a strong case for probiotic use against sepsis in preterm and term infants from developing countries. Conclusions on the value of probiotics are best based on detailed meta-analyses (MA) of randomized controlled trials (RCT). Outcomes of MA are discussed in the present review for a number of gastroenterology conditions. Since these MA pool data from trials using different probiotic species, large RCT published sometimes come to different conclusions than MA including these studies. This is not necessarily a contradiction but may only mean that the specific probiotic species did not work under the specified conditions. Positive or negative generalization about probiotics and prebiotics should be avoided. Credible effects are those confirmed in independent trials with a specified probiotic strain or chemically defined prebiotic in a specified patient population under the specified treatment conditions. Even distinct technological preparations of the same probiotic strain might affect clinical outcomes if they alter bacterial surface structures. Underpowered clinical trials are another problem in the probiotic field. Data obtained with sophisticated omics technologies, but derived from less than ten human subjects should be interpreted with caution even when published in high impact journals.

## Introduction

Probiotics are a controversial issue. Whereas, at one extreme, some scientists attribute too many effects for probiotics by claiming that they are responsible for the obesity epidemic, other scientists, at the other extreme, think that probiotics do not contribute more health benefits than eating yoghurt. Although some popular websites handle probiotics as magic bullets, many scientists deplore the contradictory evidence for the health benefits of probiotics. The economic importance of probiotics is high: The global market for probiotics amounted to $40 billion in 2017 and is predicted to increase to $64 billion by 2023 ^1^.

Part of the controversy over probiotics is due to a lack of precise definitions and the importance of details. Let us start by defining probiotics as “live microorganisms that when administered in adequate amounts confer a health benefit to the host” ^2^ and prebiotics as “a substrate that is selectively utilized by host microorganisms conferring a health benefit” ^3^. Synbiotics are dietary food supplements combining probiotics with prebiotics that support the chosen probiotic. Probiotics have been explored in an exponentially increasing number of clinical trials for their treatment effects ^4^ but are also of substantial scientific interest for understanding their mechanism of action and interaction with the microbiome. The vast probiotic literature is not easily summarized in a short review. Care for detail is needed to reach conclusions, making the writing and reading of such a review a difficult task. I will limit this overview to recent randomized clinical trials (RCTs) and meta-analyses (MAs) for a few well-investigated disease states.

Drawing conclusions from the literature in this field is difficult for several reasons. First, the probiotic literature is spread over many different medical conditions (covering health and disease), branches of medicine (from pediatrics to gerontology), and types of countries (from developing to industrialized). Second, the literature covers many different probiotic strains used at different doses and in different formulations such that rarely more than two clinical trials were conducted with the same probiotic strain against the same medical condition. This fact illustrates the difficulty of drawing conclusions from RCTs with probiotics compared with drug trials where a defined chemical compound is tested for its efficacy. This difference also explains why the European Society for Paediatric Gastroenterology, Hepatology and Nutrition (ESPGHAN) made few recommendations on specific probiotics ( *Lactobacillus rhamnosus* GG [LGG] and *Saccharomyces boulardii*) used against acute gastroenteritis (AGE) ^5^ and against antibiotic-associated diarrhea (AAD) while no optimal probiotic strain could be identified for preterm infants despite more than 11,000 subjects enrolled in RCTs ^6^. Third, many trials have methodological problems or are underpowered. Conclusions therefore rest largely on MAs. Some MAs come to positive conclusions for the efficacy of probiotics, whereas large individual trials that are part of the MA come up with negative conclusions, indicating that the specific probiotic strain(s) had no effect against the investigated condition. Overstretched negative or positive conclusions from RCTs with probiotics are to be avoided; the conclusion applies only to the specific probiotic tested against the specified clinical conditions.

## Diarrhea

### Treatment of acute gastroenteritis

In the most recent Cochrane Review, 63 studies with 8000 mostly pediatric patients ^7^ and many different probiotics were evaluated. Despite some variability, the MA showed a statistically significant effect on the primary outcome: duration of diarrhea was reduced by a mean of 24 hours compared with placebo. Since no adverse events were observed, the authors concluded that probiotics could be added to rehydration solutions for the treatment of diarrhea. However, specific probiotic regimens in defined groups of children could not be given. Although the trials included in this MA had methodological limitations (small sample sizes, questionable randomization, and blinding), ESPGHAN still formulated a recommendation ^5^.

The following two large trials challenge recommendations for LGG in AGE and raise the issue of strain-specific probiotic effects ^6, 8, 9^ or alteration of LGG by technological processing leading to the loss of pili ^10^. A multicenter Canadian RCT randomly assigned children hospitalized with AGE to probiotic treatment with a commercial mixture of *L. rhamnosus* and *Lactobacillus helveticus* versus placebo ^11^. Duration and severity of diarrhea and vomiting did not differ between the two groups. Stability of the probiotic product was confirmed. However, the *L. rhamnosus* strain used in the trial differed from the LGG reference strain by displaying different pili genes. A similar multicenter RCT randomly assigned 940 US children to LGG or placebo ^12^. Moderate to severe diarrhea developed in 12% and 13% of the probiotic and placebo patients, respectively. No significant difference in frequency or duration of diarrhea and vomiting was observed between the two groups. About 40% of the patients showed no enteropathogen in the stool. Even very-well-performed trials published in high-impact journals, like the two trials, are not beyond criticism. For example, the probiotic treatment started late in the trial ^11, 12^ such that treatment effects on acute diarrhea could not be expected.

Another MA of six RCTs (representing 1300 children cumulatively) concluded that *Bacillus clausii* significantly reduced the duration of diarrhea and hospital stay compared with controls ^13^, but strain identities were not documented. However, the largest and best controlled of the individual studies showed no effect for a mixture of four *B. clausii* strains (O/C84, N/R84, T84, and SIN84) ^14^ whereas a recent Indian trial with *B. clausii* UBBC-07 spores showed a significant but small reduction in diarrhea duration over placebo (76 versus 82 hours) ^15^.

### Prevention of acute gastroenteritis

There is a paucity of evidence for the prevention of diarrhea by probiotics in the community ^16^. In a multicenter clinical trial, more than 400 infants were randomly assigned to a control formula or a test formula containing prebiotic bovine milk oligosaccharides and the probiotic *Bifidobacterium lactis*; 60 breastfed infants served as a reference group. Diarrhea incidence and incidence of any form of infection measured over the first year of life did not differ significantly between the three groups of children ^17^ despite fecal increase of bifidobacteria in infants receiving the test formula ^18^. The control group showed a lower-than-expected diarrhea rate and thus the study was underpowered. In diarrhea stools, few enteropathogens were identified, therefore suggesting a high percentage of non-infectious diarrhea in the study. However, an RCT with *L. reuteri* DSM 17938 in 340 children reported a significant reduction in diarrheal and respiratory infections over a 6-month follow-up period ^19^.

### Nosocomial diarrhea

ESPGHAN analyzed data from eight RCTs conducted with different probiotics (LGG, DSM 17938, *B. lactis* BB-12, *B. bifidum*, two dairy strains) for the prevention of nosocomial diarrhea in 2200 children ^20^. Overall, no significant reduction was seen with probiotics over placebo for risk of nosocomial diarrhea, rotavirus diarrhea, or rotavirus shedding. When individual probiotics were evaluated, only *Lactobacillus* LGG (two RCTs, 800 children) showed a reduction of nosocomial diarrhea from 14% in controls to 5% in treated children, but no effect on rotavirus diarrhea was detected ( Table 1).

**Table 1.** Effect of different probiotic strains on nosocomial diarrhea.

Strain	Nosocomial diarrhea	Rotavirus diarrhea	Rotavirus shedding
*Lactobacillus rhamnosus* GG	+	−	−
*Lactobacillus reuteri* DSM 17938	−	−	NT
*Bifidobacterium animalis* BB-12	−	−	NT
*Bifidobacterium bifidum,* *Streptococcus thermophilus*	(+)	−	+
*Lactobacillus delbrueckii* H2B20	−	−	NT

+, significant effect; (+), positive trend; −, no effect; NT, not tested.

### Antibiotic-associated diarrhea

AAD affects about one third of children treated with antibiotics, which are the most prevalent prescription drugs in pediatrics. An MA of 23 RCTs comprising nearly 4000 patients described a reduction of AAD from 19% in placebo to 8% in probiotic-treated patients ^21^. The ESPGHAN working group for probiotics/prebiotics recently questioned the practice of pooling different probiotic strains together for MAs ^6^ and suggested that probiotic effects against AAD may be strain-specific ^22^ or even depend on the probiotic preparation technique (LGG with or without pili; ^10^). Only two probiotics—LGG and *S. boulardii*—were tested in more than one RCT. They both reduced the rate of AAD to 10% and 9% (respectively) compared with 23 to 21% in corresponding placebo controls. The quality of evidence was moderate. More recently, *Lactobacillus plantarum* LP299V treatment versus placebo was tested in 440 children receiving antibiotics. No difference was observed between the groups for incidence of AAD or watery stools ^23^. Likewise, 250 children treated with antibiotics and randomly assigned to DSM 17938 or placebo did not show a difference in AAD or diarrhea occurrence ^24^.

***Overdrawn conclusions of small studies.*** A word of caution is in place with respect to overdrawn conclusions. For example, a recent *Cell* article reported that eight human volunteers who received probiotics after antibiotic treatment and gut cleansing showed reduced gut microbiota diversity and delayed return to pre-intervention microbiome composition compared with seven controls ^25^. This observation was used on the journal’s website to warn against adverse effects of probiotics after antibiotic application. This conclusion ignores data from several studies: of probiotic versus placebo use in nearly 400 mother–child pairs where probiotics corrected undesired microbiota changes caused by antibiotic use or caesarian section ^26^; the reduction in IgE-associated allergic diseases in 140 caesarian-delivered children treated with probiotics versus controls ^27^; and again in a smaller study describing reduced diarrhea in antibiotic-treated *Clostridium difficile* patients by probiotics ^28^, where probiotics did not decrease microbial diversity compared with controls ^29^. The probiotics and microbiome areas are not fields to draw conclusions from a single study with few subjects, even though it was conducted by an impressive array of omics-technologies.

### *Clostridium difficile* infection

*C. difficile* infection (CDI) causes half a million infections and 30,000 deaths per year in the US. Since antibiotic treatment is a major risk factor for CDI and fecal microbiota transfer is an efficient treatment method, probiotics might be expected to prevent CDI when given as an adjunct to antibiotic treatment. Indeed, a recent MA of 19 studies comprising 6300 patients showed a significant CDI incidence reduction from 3.9% in the control to 1.6% in the probiotic group ^30^. However, not all studies included in this MA showed efficacy. For example, one large study including 3000 patients found no difference between probiotic ( *Lactobacillus acidophilus*, *B. bifidum*, *B. lactis*, 6 × 10 ^10^ bacteria per day for 21 days given between antibiotic doses) and placebo for any endpoint ^31^. Shen *et al*. ^30^ argued that the observed failure might be due to a too-late probiotic application compared with the start of antibiotic treatment and support their conclusion by a regression analysis showing an erosion of the probiotic effect with delayed onset. Allen *et al*. ^31^ observed only a 1% *C. difficile* diarrhea (CDD) rate in their study, whereas other studies reported CDD rates of up to 40%. Therefore, patient characteristics might influence trial outcomes, and of course not all strains or strain combinations are going to work, thus reiterating the importance of strain-specific probiotic effects.

### Traveler’s diarrhea

Traveler’s diarrhea (TD) is a common condition affecting adults traveling to developing countries and is associated with bacterial pathogens like enterotoxigenic *Escherichia coli*. The 2017 guidelines of the International Society for Travel Medicine concluded that there is insufficient evidence to recommend prebiotics or probiotics for the prevention or treatment of TD. Older data showed a reduction in TD incidence from 43% (placebo) to 32% in Austrian tourists taking a high dose of *S. boulardii* ^32^. A recent MA of 11 trials described a small but significant effect of interventions ^33^. However, the effect was mediated by prebiotics while probiotics showed no effect ^33^. In another study, 330 travelers were randomly assigned to a commercial galacto-oligosacchride (GOS) prebiotic or placebo. Diarrhea incidences were 19% and 29%, respectively. GOS prevented mild, one-day diarrhea but had no effect on the severity or duration of diarrhea, and the effect became visible only after one week of prebiotic treatment ^34^. Like probiotic strains effects, prebiotic effects should be specified according to distinct chemical characteristics of the prebiotics.

## Other gastroenterology disorders

### Infant colic

Let us consider one example in more detail: infant colic or excessive crying without obvious cause. Effects of a gut microbiota disturbance on the immune or intestinal nervous system were hypothesized as possible causes for these occurrences and have motivated probiotic intervention trials. An MA of four RCTs with *L. reuteri* DSM 17938 showed a statistically significant reduction of daily crying time from 160 (placebo) to 140 (probiotic) minutes in breastfed but not in formula-fed infants ^35, 36^. Since the two groups of infants differed in gut microbiota composition, interaction of *L. reuteri* with the resident microbiota was suspected to influence the probiotic effect ^37^.

A recent small trial in breastfed infants confirmed this observation with a greater effect size by halving the crying time ^38^. A mixture of eight different probiotics resulted in a reduction of the daily crying time from 98 (placebo) to 68 (probiotic) minutes in another small trial ^39^. Also, in a larger trial comparing two lactobacilli plus the prebiotic fructo-oligosaccharide (FOS) against placebo, a significant reduction in crying time was found in breastfed infants ^40^. A Cochrane Review ^41^ found no evidence that probiotics ( *L. reuteri*, *L. rhamnosus*, *Lactobacillus paracasei*, and *Bifidobacterium animalis*) are more effective than placebo in preventing infantile colic, although crying time was reduced.

### Radiation diarrhea

Gastroenterology problems are the most obvious targets for oral probiotics, and although many conditions have been explored, the evidence level is still mixed ^42^. Here, only brief hints to recent literature references are given. An MA on probiotics against radiation therapy–induced diarrhea ^43^, which evaluated six RCTs and 900 patients, found a lower incidence of diarrhea in the probiotic versus the placebo group but without observing a sparing effect on frequency and duration of anti-diarrhea medication use and stool consistency.

***Inflammatory bowel disease.*** In one MA of probiotic interventions, no benefits of probiotics were found for inducing remission of active Crohn’s disease (CD), preventing relapse of quiescent CD, or in surgically induced remission of CD ^44^. Another MA reported some beneficial effects in reaching remission from ulcerative colitis (UC), but the effects depended on the UC scale adopted and inclusion of bifidobacteria ^45^.

***Helicobacter pylori.*** Two MAs on probiotics as adjuncts to antibiotics differed on their conclusion for the eradication of *Helicobacter pylori* infection, but both analyses reported a reduced incidence of antibiotic-associated adverse side effects ^46, 47^.

**Irritable bowel syndrome.** In a trial enrolling 113 celiac disease patients who displayed irritable bowel syndrome (IBS) despite a gluten-free diet, probiotic intervention with a mixture of *Lactobacillus casei*, *L. plantarum*, *B. animalis*, *B. lactis*, and *Bifidobacterium breve* improved IBS symptoms over placebo. However, the improvement was not maintained after the cessation of treatment ^48^. Fifty-three trials enrolling 5500 patients were evaluated for the effect of different probiotics or probiotic combinations against symptoms in patients with IBS. Although some probiotics showed effects ( *Lactobacillus* on flatulence and *Bifidobacterium* on abdominal pain), it remained unclear which probiotic combination, species, or strain should be preferred in the individual patient ^49^.

## Healthy adults

For regulatory reasons, probiotics are frequently marketed with claims such as boosting immunity or restoring microbiota balances. This raises the question of whether probiotics have an effect in healthy adults, but one should be aware that the definition of health is not precise in the medical literature ^50^. An analysis of the literature revealed that interventions with *L. plantarum* and *L. casei* or *Lactobacillus gasseri*, *B. longum*, and *B. bifidum* as well as with yoghurt starters *Lactobacillus delbrueckii* and *Streptococcus thermophilus* reduced the incidence, duration, and symptoms of common cold infections but not of influenza ^51^. However, a separate MA showed a stimulation of influenza vaccination by probiotics ^52^. Several studies also reported a better gut comfort (improvement in bowel movement, defecation frequency, and stool consistency) ^51^. One might add that reduced use of antibiotics against respiratory infections that are likely of viral origin and annual influenza vaccination will undoubtedly have a beneficial effect on the microbiota balance.

## Sepsis and necrotizing enterocolitis

Late-onset sepsis is a major cause of morbidity and mortality in preterm infants from industrialized and developing countries. Rao *et al*. ^53^ conducted an MA of probiotic use against late-onset sepsis in preterm infants including 37 RCT enrolling 9400 infants. The authors described a significant reduction of late-onset sepsis from 16.3% in placebo to 13.9% in probiotic recipients. The difference remained significant if analyzed in infants treated with lactobacilli, bifidobacteria, or single or multiple probiotics. A significant reduction of late-onset sepsis and death was also seen in probiotic-treated preterm infants from developing countries ^54^.

Necrotizing enterocolitis (NEC) is another severe disease of preterm infants. An MA of 42 RCTs found a significant reduction of NEC and mortality in infants treated with probiotic versus placebo ^55^. Recent studies investigated species-specific effects and reported prevention of severe NEC with *B. breve* and *B. lactis* ^56, 57^, but probiotics had no effect on the relative risk of surgery for NEC ^58^. Multiple-strain probiotics used at high dosage were most effective ^59^. Infants treated with a probiotic mix of *Bifidobacterium infantis*, *B. lactis*, and *S. thermophilus* showed a reduction of NEC from 4.4% (placebo) to 2% (probiotic) ^60^, whereas infants treated with *B. breve* showed no effect on NEC or death ^61^.

The authors of MAs ^8, 9, 53^ made a strong case for the use of probiotics in preterm infants, arguing that no other interventions showed effects at the low cost of probiotics ($1 per day) ^62^. A small (2%) reduction in late-onset sepsis or NEC will make a clinically important difference when neonatal infections are responsible for a quarter of the one million neonatal deaths occurring every year in, for example, India. The authors rejected the argument that different probiotics cannot be pooled in MA. They also argued that some large negative trials were closer to being “inconclusive” than “negative”.

A probiotic effect was also seen in an Indian study with 4500 newborns who were randomly assigned to a synbiotic treatment ( *L. plantarum* plus FOS) or placebo. The treatment resulted in a striking 40% reduction of sepsis, which remained significant for culture-confirmed sepsis ^63^. Notably, the study showed a significant reduction of lower respiratory tract infections necessitating antibiotic use and of diarrhea, local infections, and omphalitis. The authors attributed this striking anti-infectious effect to the superior ability of the probiotic to colonize the infant gut. However, the death rate in the infants was not affected.

## Future trends

Each year, about 230 million surgical interventions are performed worldwide; some lead to post-operative complications consisting of surgical site infections, urinary infections, pneumonia, and sepsis. Preventive antibiotic treatment is standard but is complicated by rising antibiotic resistance. A recent network MA of 2952 abdominal surgery patients from 31 studies demonstrated beneficial effects of synbiotics against surgical site infection ^64^. Synbiotics (successful trials used mostly *L. plantarum*, *L. casei*, and *B. breve* combined with GOS) were also the best interventions to reduce pneumonia, urinary infection, sepsis, hospital stay, and antibiotic use but had no effect on mortality ^64^. These authors concluded that surgeons should consider the use of synbiotics as an adjunctive therapy to prevent post-operative complications. Another MA documented the same effects ^65^ but concluded that the observations should be interpreted with caution because of a possible publication bias. Two RCTs were recently added to the list of clinical trials. Fifty-five liver transplantation patients received a four-strain probiotic or placebo before the scheduled transplantation. At 90 days after intervention, the infection rate was strikingly lower in probiotic recipients compared with controls (5% versus 48%, *P* = 0.002) ^66^. In contrast, no synbiotic effect with *L. paracasei*, *L. rhamnosus*, *L. acidophilus*, and *B. lactis* plus FOS was seen in 18 head-and-neck cancer patients treated post-operatively compared with 18 placebo-treated controls ^67^.

Healthy children receiving human milk oligosaccharides (HMOs) showed a lower rate of bronchitis compared with controls ^68^. Several clinical trials with HMOs are registered, and HMOs will likely represent a new trend for prebiotics. Post-biotics—chemically defined metabolites or cell wall compounds released by probiotics—could also gain importance in the future ^69^. Likewise, probiotics are increasingly explored for application in atopic diseases and skin health as well as vaginal preparations against bacterial vaginosis and associated infection problems ^70^. Probiotics might influence the gut–brain axis and thus influence IBS, mood disorders, and anxiety.

## Conclusions

The value of human microbiome-based products is currently estimated to be about $400 million worldwide and is expected to reach $1 billion over the next five years ^71^. Despite the coverage of microbiome research in high-impact scientific journals, the derived products represent a small fraction of the market value of probiotic products. Consumer and industrial interest in probiotics are thus very high, but it should be stressed that probiotics are not magic bullets. Generalizations about “probiotics” should be avoided. If something is linked with medical effects, these are specific strains or specific prebiotic compounds providing a specific health effect in a particular patient group from a particular population. As trivial as this statement is, it is still very difficult to distill these positive conclusions from a complex and sometimes seemingly contradictory research literature. The Human Microbiome Project has already reached entrance into the 2010 edition of Harrison’s *Principles of Internal Medicine* ^72^ before the first products of microbiome research appeared at the bedside, while probiotics are found only as small scattered notes in the same standard medical textbook. It is time for the medical curriculum to portray microbes not only as pathogens causing disease but also as important ingredients of human health. Literacy of the next generation of doctors in microbiome and probiotic research is desirable. Microbiologists and clinicians should help in this endeavor by research substantiating specific claims for specific probiotics in specific patients.
